# Topical Osmoprotectant for the Management of Postrefractive Surgery-Induced Dry Eye Symptoms: A Randomised Controlled Double-Blind Trial

**DOI:** 10.1155/2018/4324590

**Published:** 2018-02-20

**Authors:** R. M. Hazarbassanov, N. G. T. Queiroz-Hazarbassanov, J. N. Barros, J. A. P. Gomes

**Affiliations:** ^1^Department of Ophthalmology and Visual Sciences, Paulista Medical School, Federal University of São Paulo, São Paulo, SP, Brazil; ^2^Applied Pharmacology and Toxicology Laboratory, Veterinary Pathology Department, School of Veterinary Medicine, University of São Paulo, São Paulo, SP, Brazil

## Abstract

**Background:**

Dry eye disease (DED) is one of the most common complications following refractive surgery.

**Purpose:**

Evaluate the efficacy of an osmoprotective eye drop (Optive®) for the management of induced DED in refractive surgery patients.

**Design:**

Double-masked randomised controlled trial.

**Methods:**

Twenty-two refractive surgery patients oriented to apply FreshTears (FT; *n* = 13) or Optive (Op; *n* = 9), topically, QID, for 3 months. Eye exams were performed before surgery (T0) and 1-month (T1) and 3-month (T3) follow-up and consisted of tear film osmolarity, Schirmer 1 test, tear film breakup time (TBUT), fluorescein staining, and ocular surface disease index (OSDI) and patient symptoms questionnaires.

**Main Outcome Measures:**

Pain and osmolarity.

**Results:**

Pain increased significantly for FT at T3 (*p* < 0.05). A reduction in osmolarity was observed at T1 and T3 for Op group (*p* < 0.01) and at T3 for FT group (*p* < 0.05). TBUT showed a decrease between T0 and T1 for FT (*p* < 0.05). Schirmer 1 values increased significantly for Op in T1.

**Conclusions:**

Op was superior to FT in regard to pain, osmolarity, TBUT, and Schirmer 1. Osmoprotectant solutes, such as L-carnitine, could attenuate inflammation and secondary DED. Osmoprotective lubricants can be effectively applied for the prevention of refractive surgery-related dry eye symptoms and signs.

## 1. Introduction

Dry eye disease (DED) is a complex and multifactorial disease, which is reported as a complication in 40 to 60% of refractive surgery patients [1–3]. Reduced corneal sensation was proposed as the basis of reduced blinking and tear secretion after laser-assisted in situ keratomileusis (LASIK) surgery, and both can contribute to a state of aqueous deficiency [4]. Additionally, it was proposed that such symptomatic condition is due to disruption of trophic sensory input to the denervated region. This was denominated LASIK-induced neuroepitheliopathy (LINE) [5]. A similar situation may occur subsequent to photorefractive keratectomy (PRK) [6]. Postoperative discomfort has been described as a drawback of PRK, hence requiring pain and discomfort management with topical NSAIDs, gabapentin, oxycodone and acetaminophen, diclofenac, or others [7].

The use of nonpreserved artificial tears and other lubricants has been suggested as useful for the treatment of dry eye symptoms and for reducing the impact on goblet cell density after LASIK [8]. The few studies that to date have investigated tear osmolarity after LASIK have found that osmolarity drops immediately after surgery and increases significantly and remains markedly higher for at least 6 months [9–11]. Tear film hyperosmolarity activates MAP kinases and NF-*κ*B signalling pathways in ocular surface epithelial cells [12, 13] and the generation of inflammatory cytokines [14]. Therefore, the purpose of this study was to verify the therapeutical effect of an osmoprotective eye drop (Optive) for the management of induced aqueous deficient DED in patients subjected to refractive surgery.

## 2. Patients and Methods

This research protocol was approved according to the Ethics Committee in Research, UNIFESP, under number 1346/08 and registered at http://ClinicalTrials.gov (ID number NCT01741987). The visits were established before surgery (T0) and 1-month (T1) and 3-month (T3) posttreatment with osmoprotective and nonosmoprotective lubricants.

Twenty-two patients were selected from the Refractive Surgery Department, UNIFESP, who were referred for bilateral LASIK (11 patients) or PRK (11 patients). Patient number was calculated by previous pilot study with pain as the primary outcome, in which 100% Op patients reported improvement after 3 months. LASIK flap insertion position was superior and performed with Moria™ microkeratome. The flap was 9 mm in diameter and 130 *μ*m thick. The refractive surgery applied excimer argon fluoride laser (193 nm) (LADAR Vision 4000, Alcon). A suspension of topical steroid and antibiotic (moxifloxacin 0.5% and dexamethasone phosphate 0.1%) was prescribed postoperatively for patients QID for 1 week (LASIK) and 2 weeks (PRK). Consecutively, thirteen patients were randomised by an online random allocation tool to receive topical administration QID of FreshTears (FT, Allergan Inc.) (6 LASIK and 7 PRK) while nine patients were given topical administration QID of Optive (Op, Allergan Inc.) (5 LASIK and 4 PRK). Labels were removed, and both drops were repackaged in dark plastic bags in order to mask the brands to patients and principal investigator.

Optive contains sodium carboxymethylcellulose (CMC), glycerine, erythritol, and stabilised sodium chlorite complex (Purite™). The main osmoprotectant component is L-carnitine, and its osmolarity is 328 mOsm/L [15]. FreshTears contains CMC, sodium chloride, potassium chloride, calcium chloride dihydrate, magnesium chloride hexahydrate, boric acid and sodium borate decahydrated (as buffering agents), purified water, and Purite. The osmolarity of FT is 280 mOsm/L.

The subjects were submitted to the following tests, exactly in the order cited, during the first visit (T0) and at follow-up visits (T1 and T3): best spectacle-corrected visual acuity (BSCVA) converted for LogMar scale, tear film osmolarity by electrical conductivity [16], biomicroscopy [17], Schirmer 1 test without anesthesia [18], tear film breakup time (TBUT) [18], fluorescein staining [19], completing patient's symptoms questionnaire and ocular surface disease index (OSDI) [20], lissamine green staining [18], and impression cytology (IC) and staining by periodic acid Schiff-hematoxylin (PAS-H). Total IC scores were defined as a sum of scores for each morphological change, such as cellularity, cohesivity, nuclear/cytoplasm ratio, snake-like chromatin, goblet cell density, and inflammation [21]. Consecutively, delta IC total scores were calculated by the difference between T0 scores and T3 total scores. Reported pain and osmolarity data were regarded as the primary outcome measures.

Safety parameters were assessed through eye exams and observation of adverse events throughout the study. If an adverse event was severe or caused impact to the patients' life quality, treatment would be interrupted.

Continuous data distribution was analysed by Kolmogorov-Smirnov normality test. Values were represented by sample mean and standard deviation or standard error of the mean (SEM). Baseline demographical and ophthalmological data were analysed by Student's unpaired *t*-test when parametric and Mann-Whitney test when nonparametric. When more than 2 samples and 2 periods were compared, the repeated measures ANOVA with Tukey posttest was applied. Nonparametric data were represented by inferior and superior quartile median, with Wilcoxon comparison when 2 periods were analysed and Friedman when more than 2 periods were analysed. A *p* value of less than 0.05 was accepted as statistically significant. GraphPad Prism version 5 was used for statistical analyses.

## 3. Results

Baseline demographical and ophthalmological examination data are summarised in Table 1. There was no statistically significant difference between groups for the evaluated parameters.

### 3.1. OSDI and Symptoms Questionnaire

After refractive surgery was performed, patient maintenance started with FT and Op administration and evaluated at 1- and 3-month follow-up. A comparison of the mean OSDI scores did not reveal any changes in the evaluated periods (repeated measures ANOVA, *p* > 0.05).

**Figure 1:**
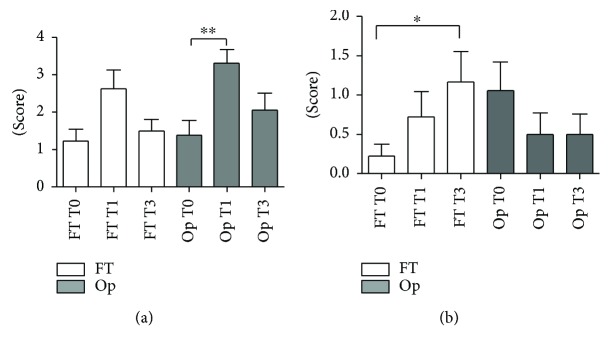
Mean and SEM values of questionnaires scores compared between FT and Op groups before surgery (T0) and 1 month (T1) and 3 months (T3) after treatment. (a) Dryness scores. The score is significantly higher for Op group in T1 compared to T0 (Friedman test, ^∗∗^*p* < 0.01). (b) Pain scores. There was a significant increase between T0 and T3 for the FT group (Friedman test, ^∗^*p* < 0.05).

In regard to symptoms questionnaire, Op-treated patients reported more dryness at T1 visit (Friedman test, *p* < 0.01), though both treatments showed a return to baseline values at T3 (Figure 1(a)). The symptom of pain increased significantly for the FT group between T0 and T3 (Friedman test, *p* < 0.05) but tended to be lower than baseline in the Op group for T1 and T3 (Figure 1(b)). However, the symptoms of burning (ANOVA-Tukey, *p* > 0.05), foreign body sensation and blurred vision (Friedman, *p* > 0.05), and photophobia and sum of all symptoms (repeated measures ANOVA, *p* > 0.05) showed no difference between treatments for all periods.

### 3.2. Ophthalmological Exams

Visual acuity, as evaluated by LogMar scale, did not present any difference between treatment groups, for the T1 and T3 periods (Wilcoxon, *p* > 0.05).

**Figure 2:**
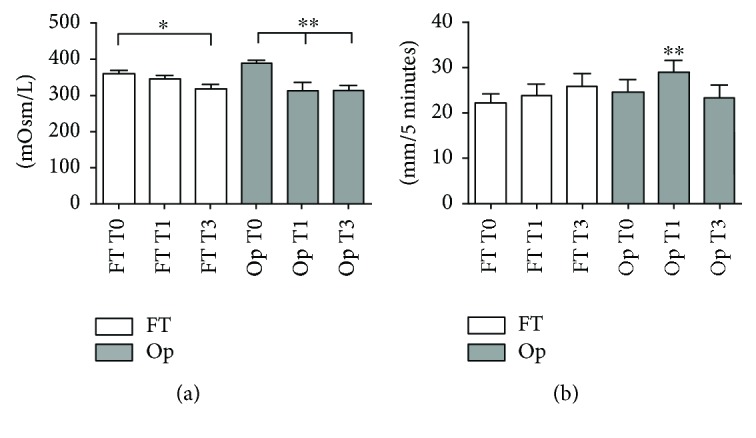
(a) Tear osmolarity mean and SEM values compared between FT and Op groups before surgery (T0) and 1 month (T1) and 3 months (T3) of treatment. FT group presented a significant reduction in osmolarity values after 3 months of treatment (^∗^ANOVA-Tukey, *p* < 0.05). In the Op group, reduced osmolarity was noted between T0 and T1 and T0 and T3 (^∗∗^ANOVA-Tukey, *p* < 0.01). (b) Schirmer 1 test mean and SEM values. An increase was observed for Op group, in T1 only (Friedman test, ^∗∗^*p* < 0.01).

A significant reduction in osmolarity values between T1 and T3 in comparison to T0 for the Op group was observed (ANOVA-Tukey, *p* < 0.01), while for the FT group, a difference was observed only between T0 and T3 (ANOVA-Tukey, *p* < 0.05) (Figure 2(a)).

It was noticed that Op significantly improved Schirmer 1 at T1 and returned to baseline values at T3 (Friedman test, *p* < 0.01) (Figure 2(b)). Additionally, the analysis of TBUT showed no statistically significant difference between periods for both groups (Friedman test, *p* > 0.05).

Considering vital stains, no alteration was observed for lissamine green staining (repeated measures ANOVA, *p* > 0.05) and fluorescein (Friedman test, *p* > 0.05).

### 3.3. Impression Cytology

**Figure 3:**
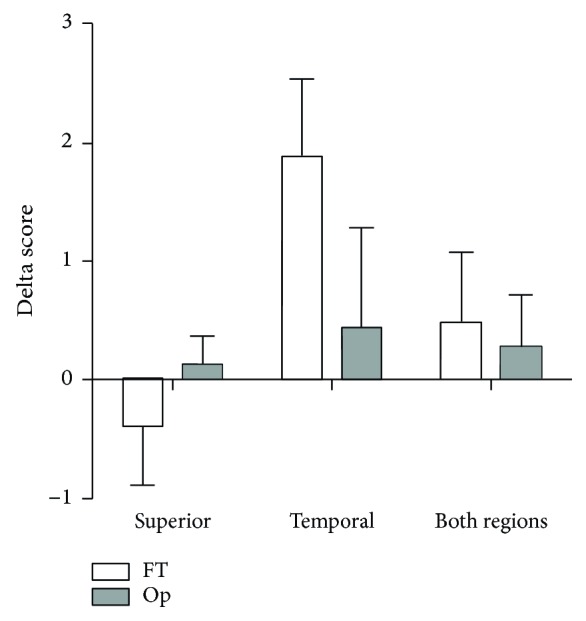
Mean and SEM of impression cytology score deltas calculated by the difference between 3 months (T3) values and before surgery (T0) values. No difference was found between FT and Op treatments (Mann-Whitney, *p* > 0.05).

It was assessed whether FT and Op groups showed differences between T0 and T3 scores of ocular surface changes as shown by PAS-H staining in impression cytology samples. Delta total score analysis revealed no significant change for superior, temporal, and both regions grouped (Mann-Whitney, *p* > 0.05) (Figure 3).

## 4. Discussion

According to the Dry Eye WorkShop (DEWS) [22], LASIK-induced dry eye is a form of non-Sjögren's syndrome (SS) aqueous deficient DED. After LASIK or PRK, patients can report significant dry eye for several months, an effect that is due to the section of corneal nerves during surgery [23]. The sensory denervation of the ocular surface after bilateral LASIK disrupts the lachrymal dynamics and causes irritation symptoms [24]. Sub-basal nerves begin to recover from 3 to 6 months after surgery and are 50% of the original preoperative density within 2 years after surgery [25]. In a randomised trial, symptoms of DED apparently resolved at 1 year postoperatively, for both LASIK and PRK [6].

In the present study, patients who underwent LASIK and PRK were grouped, and according to published data, corneal sensitivity is reduced by both techniques until 3 months postoperatively [26]. Previous studies have shown that there is no significant difference in dry eye symptoms between LASIK and PRK without ocular surface management. [8]. Also, Toda et al. evaluated the effect of LASIK in patients with and without dry eye before surgery, and the recovery time of corneal sensitivity in non-dry eye patients was 3 months [27]. We did not perform esthesiometry tests because of diurnal variability of corneal sensitivity [28], and approximately 45% of patients were contact lens users (as shown in Table 1), a condition reported to reduce corneal sensitivity [24].

Evaluation of dryness showed significantly increased scores for the Op-treated patients at T1 and decreased at T3. Though not significant, this pattern was also observed for FT. This seems to point to the effect of LASIK denervation (1 month) and improvement conferred by FT and Op (3 months), possibly related to the viscoelastic properties of CMC and lubricants. Carboxymethyl cellulose-based eye drops have been widely used after LASIK to accelerate recovery of the ocular surface and to minimise symptoms [29]. It has been described that Op treatment was able to diminish symptoms such as dryness, foreign body, and burning compared to baseline score values [30]. It is noteworthy, however, that while pain score worsens significantly for FT group, Op treatment appears to reduce pain complaints, a possible anti-inflammatory effect of osmoprotection. To the authors' knowledge, this is the first report of a non-anti-inflammatory drug that can be applied for the management of postrefractive surgery pain and discomfort. Although with a relatively small patient number, pain reduction seems to be consistent and has also been observed in evaporative dry eye patients as well (unpublished data).

Additionally, refractive surgery increases tear osmolarity, with no significant difference between LASIK and LASEK [10]. Refractive surgery severs corneal nerve endings, and the loss of stimulation increases osmolarity by decreasing lacrimal gland secretion of proteins, electrolytes, and water [31]. Posttreatment osmolarity values measured in our study by electrical conductivity were similar to normal values found by Ogasawara et al. [16]. Notwithstanding, it should be remarked that lenses use can cause increased tear film osmolarity, with no association with ocular symptoms [32, 33], which could explain our elevated baseline osmolarity values. Lee et al. evaluated tear osmolarity post-LASIK and PRK and have found that it peaked after 3 months and returned to baseline values in 6 months and suggested dry eye treatment for these patients [11]. Herein, we observed a significant reduction in tear osmolarity values for Op group after 1 month postoperatively, which was sustained also after 3 months. However, FT control treatment decreased osmolarity only after 3 months, but not after 1 month, as previously reported by Benelli et al. [34]. Considering that hyperosmolarity leads to ocular surface inflammation [35], this earlier osmolarity reduction could be a result of osmoprotectant compatible solutes present in the composition of Op, such as L-carnitine [36], and thus, may attenuate inflammation and DED secondary to LASIK and PRK.

Tear secretion (Schirmer 2 with anesthesia) decreases after LASIK or LASEK and returns to preoperative levels between 1 and 6 months, when treatment with artificial tears was led up to two weeks postsurgery [10, 37, 38]. Additionally, without any treatment, Schirmer 1 values decrease 1 week after LASIK, returning to baseline in 3 months [39]. In our study, using Schirmer 1, our results have shown that while FT patients did not present any significant difference, Op treatment improved tear secretion at T1, returning to baseline in T3.

Goblet cell density has been shown to decrease after 1 week and 1 month LASIK [40] and return to preoperative levels after 6 to 9 months [39], while a reduced nucleus/cytoplasm ratio can be noticed up to 6 months after surgery. [40] We did not perform IC at 1 month postoperatively since it is a short interval to observe therapeutical effects. In our results, although not statistically significant, an increase of morphological changes total score by impression cytology in the temporal region after 3 months, for FT, was observed. To Op, however, mean scores after refractive surgery did not change, which could be justified by a possible osmoprotection effect, leading to less ocular surface damage.

Op has presented superior results than FT in regard to the parameters of pain, tear film osmolarity, and Schirmer 1, while FT and Op treatments appear to have similar therapeutic effects on dryness complaints. Eye drops which contain osmoprotectant components are interesting pharmacological resources to safely and effectively prevent refractive surgery discomfort related to dry eye symptoms and signs.

## Figures and Tables

**Table 1:** Baseline demographical and ophthalmological examination data of patients randomised into FT and Op treatments.

	FT (*n* = 13; 26)	Op (*n* = 9; 18)	*p* value
Age (years)	38.21 ± 11.52	33.00 ± 7.75	0.4425
Gender (as female %)	71.4%	54.5%	0.4108
OSDI (score)	14.48 ± 3.26	24.95 ± 5.99	0.1176
Schirmer 1 (mm/5 min)	21.22 ± 10.47	25.77 ± 10.85	0.0961
TBUT (sec)	8.18 ± 4.02	8.28 ± 3.89	0.9758
Fluorescein (score)	0.15 ± 0.36	0.32 ± 0.68	0.1644
Lissamine (score)	1.04 ± 0.61	0.82 ± 0.66	0.2342
BSCVA (LogMar)	0.002 ± 0.052	0.036 ± 0.072	0.109
Osmolarity (mOsm/L)	359.5 ± 9.52	383.3 ± 8.33	0.0781
Spherical equivalent (D)	−4.02 ± 2.8	−3.56 ± 1.7	0.5745
Contact lens users (%)	42.86%	44.44%	0.7219
Contact lens use (years)	9.2 ± 10.2	8.5 ± 4.2	0.6689

Values represent mean ± standard deviation. OSDI: ocular surface disease index; TBUT: tear film breakup time; BSCVA: best spectacle-corrected visual acuity.

## References

[1] De Paiva C. S., Chen Z., Koch D. D. (2006). The incidence and risk factors for developing dry eye after myopic LASIK. *American Journal of Ophthalmology*.

[2] Yu E. Y., Leung A., Rao S., Lam D. S. (2000). Effect of laser in situ keratomileusis on tear stability. *Ophthalmology*.

[3] Salomao M. Q., Ambrosio R., Wilson S. E. (2009). Dry eye associated with laser in situ keratomileusis: mechanical microkeratome versus femtosecond laser. *Journal of Cataract and Refractive Surgery*.

[4] Toda I., Asano-Kato N., Komai-Hori Y., Tsubota K. (2001). Dry eye after laser in situ keratomileusis. *American Journal of Ophthalmology*.

[5] Ambrosio R., Tervo T., Wilson S. E. (2008). LASIK-associated dry eye and neurotrophic epitheliopathy: pathophysiology and strategies for prevention and treatment. *Journal of Refractive Surgery*.

[6] Murakami Y., Manche E. E. (2012). Prospective, randomized comparison of self-reported postoperative dry eye and visual fluctuation in LASIK and photorefractive keratectomy. *Ophthalmology*.

[7] Woreta F. A., Gupta A., Hochstetler B., Bower K. S. (2013). Management of post-photorefractive keratectomy pain. *Survey of Ophthalmology*.

[8] Albietz J. M., SG M. L., Lenton L. M. (2003). Ocular surface management of photorefractive keratectomy and laser in situ keratomileusis. *Journal of Refractive Surgery*.

[9] Hassan Z., Szalai E., Berta A., Modis L., Nemeth G. (2013). Assessment of tear osmolarity and other dry eye parameters in post-LASIK eyes. *Cornea*.

[10] Dooley I., D'Arcy F., O'Keefe M. (2012). Comparison of dry-eye disease severity after laser in situ keratomileusis and laser-assisted subepithelial keratectomy. *Journal of Cataract and Refractive Surgery*.

[11] Lee J. B., Ryu C. H., Kim J., Kim E. K., Kim H. B. (2000). Comparison of tear secretion and tear film instability after photorefractive keratectomy and laser in situ keratomileusis. *Journal of Cataract and Refractive Surgery*.

[12] Li D. Q., Chen Z., Song X. J., Luo L., Pflugfelder S. C. (2004). Stimulation of matrix metalloproteinases by hyperosmolarity via a JNK pathway in human corneal epithelial cells. *Investigative Ophthalmology & Visual Science*.

[13] Luo L., Li D. Q., Corrales R. M., Pflugfelder S. C. (2005). Hyperosmolar saline is a proinflammatory stress on the mouse ocular surface. *Eye & Contact Lens*.

[14] De Paiva C. S., Corrales R. M., Villarreal A. L. (2006). Corticosteroid and doxycycline suppress MMP-9 and inflammatory cytokine expression, MAPK activation in the corneal epithelium in experimental dry eye. *Experimental Eye Research*.

[15] Evangelista M., Koverech A., Messano M., Pescosolido N. (2011). Comparison of three lubricant eye drop solutions in dry eye patients. *Optometry and Vision Science*.

[16] Ogasawara K., Mitsubayashi K., Tsuru T., Karube I. (1996). Electrical conductivity of tear fluid in healthy persons and keratoconjunctivitis sicca patients measured by a flexible conductimetric sensor. *Graefe's Archive for Clinical and Experimental Ophthalmology*.

[17] Behrens A., Doyle J. J., Stern L. (2006). Dysfunctional tear syndrome: a Delphi approach to treatment recommendations. *Cornea*.

[18] Pflugfelder S. C., Tseng S. C., Sanabria O. (1998). Evaluation of subjective assessments and objective diagnostic tests for diagnosing tear-film disorders known to cause ocular irritation. *Cornea*.

[19] Afonso A. A., Monroy D., Stern M. E., Feuer W. J., Tseng S. C., Pflugfelder S. C. (1999). Correlation of tear fluorescein clearance and Schirmer test scores with ocular irritation symptoms. *Ophthalmology*.

[20] Schiffman R. M., Christianson M. D., Jacobsen G., Hirsch J. D., Reis B. L. (2000). Reliability and validity of the Ocular Surface Disease Index. *Archives of Ophthalmology*.

[21] Murube J., Rivas L. (2003). Impression cytology on conjunctiva and cornea in dry eye patients establishes a correlation between squamous metaplasia and dry eye clinical severity. *European Journal of Ophthalmology*.

[22] DEWS (2007). The definition and classification of dry eye disease: report of the Definition and Classification Subcommittee of the International Dry Eye WorkShop (2007). *The Ocular Surface*.

[23] Ang R. T., Dartt D. A., Tsubota K. (2001). Dry eye after refractive surgery. *Current Opinion in Ophthalmology*.

[24] Battat L., Macri A., Dursun D., Pflugfelder S. C. (2001). Effects of laser in situ keratomileusis on tear production, clearance, and the ocular surface. *Ophthalmology*.

[25] Kaufman S. C., Kaufman H. E. (2006). How has confocal microscopy helped us in refractive surgery?. *Current Opinion in Ophthalmology*.

[26] Perez-Santonja J. J., Sakla H. F., Cardona C., Chipont E., Alio J. L. (1999). Corneal sensitivity after photorefractive keratectomy and laser in situ keratomileusis for low myopia. *American Journal of Ophthalmology*.

[27] Toda I., Asano-Kato N., Hori-Komai Y., Tsubota K. (2002). Laser-assisted in situ keratomileusis for patients with dry eye. *Archives of Ophthalmology*.

[28] Millodot M. (1972). Diurnal variation of corneal sensitivity. *The British Journal of Ophthalmology*.

[29] Lenton L. M., Albietz J. M. (1999). Effect of carmellose-based artificial tears on the ocular surface in eyes after laser in situ keratomileusis. *Journal of Refractive Surgery*.

[30] Davitt W. F., Bloomenstein M., Christensen M., Martin A. E. (2010). Efficacy in patients with dry eye after treatment with a new lubricant eye drop formulation. *Journal of Ocular Pharmacology and Therapeutics*.

[31] Gilbard J. P., Rossi S. R. (1990). Tear film and ocular surface changes in a rabbit model of neurotrophic keratitis. *Ophthalmology*.

[32] Iskeleli G., Karakoc Y., Aydin O., Yetik H., Uslu H., Kizilkaya M. (2002). Comparison of tear-film osmolarity in different types of contact lenses. *The CLAO Journal*.

[33] Sarac O., Gurdal C., Bostanci-Ceran B., Can I. (2012). Comparison of tear osmolarity and ocular comfort between daily disposable contact lenses: hilafilcon B hydrogel versus narafilcon A silicone hydrogel. *International Ophthalmology*.

[34] Benelli U., Nardi M., Posarelli C., Albert T. G. (2010). Tear osmolarity measurement using the TearLab Osmolarity System in the assessment of dry eye treatment effectiveness. *Contact Lens & Anterior Eye*.

[35] Pflugfelder S. C., de Paiva C. S., Tong L., Luo L., Stern M. E., Li D. Q. (2005). Stress-activated protein kinase signaling pathways in dry eye and ocular surface disease. *The Ocular Surface*.

[36] Peluso G., Barbarisi A., Savica V. (2000). Carnitine: an osmolyte that plays a metabolic role. *Journal of Cellular Biochemistry*.

[37] Donnenfeld E. D., Solomon K., Perry H. D. (2003). The effect of hinge position on corneal sensation and dry eye after LASIK. *Ophthalmology*.

[38] Vroman D. T., Sandoval H. P., Fernandez de Castro L. E., Kasper T. J., Holzer M. P., Solomon K. D. (2005). Effect of hinge location on corneal sensation and dry eye after laser in situ keratomileusis for myopia. *Journal of Cataract and Refractive Surgery*.

[39] Konomi K., Chen L. L., Tarko R. S. (2008). Preoperative characteristics and a potential mechanism of chronic dry eye after LASIK. *Investigative Ophthalmology & Visual Science*.

[40] Rodriguez-Prats J. L., Hamdi I. M., Rodriguez A. E., Galal A., Alio J. L. (2007). Effect of suction ring application during LASIK on goblet cell density. *Journal of Refractive Surgery*.

